# Preparation and In Vitro Bioactivity Study of a Novel Hollow Mesoporous Bioactive Glass Nanofiber Scaffold

**DOI:** 10.3390/molecules27227973

**Published:** 2022-11-17

**Authors:** Jian Xiao, Qianghua Wei, Jinhong Xue, Zide Yang, Zhicheng Deng, Fulai Zhao

**Affiliations:** 1 School of Physics and Electronic Information, Shangrao Normal University, Shangrao 334001, China; 2 School of Materials Science and Engineering, Tianjin University, Tianjin 300072, China; 3 Primary Health Department, Shangrao Health Committee, Shangrao 334001, China; 4 Basic Department, Jinci College of Shanxi Medical University, Taiyuan 030025, China; 5 Institute of Science and Technology, China Railway Nanchang Group Co., Ltd., Nanchang 330002, China

**Keywords:** sol-gel method, three-dimensional, hollow, mesoporous bioactive glass nanofiber scaffold, bioactivity

## Abstract

In this study, a novel three-dimensional hollow mesoporous bioactive glass nanofiber scaffold has been synthesized with a template-assisted sol-gel method using bacterial cellulose (BC) as a template and nonionic triblock copolymer (P123) as a pore-directing agent, ethyl orthosilicate (TEOS), calcium nitrate tetrahydrate (CN), and triethyl phosphate (TEP) as glass precursors. Scanning and transmission electron microscopies, X-ray diffraction, nitrogen adsorption-desorption, and nuclear magnetic resonance method were applied to characterize the morphology, crystal structure, and chemical structure of the mesoporous bioactive glass nanofiber scaffold. Furthermore, the in vitro bioactivity and biocompatibility were also explored. The obtained scaffold depicted nanofiber-like morphology and interconnected three-dimensional network structure that replicated the BC template. The scaffold showed a large specific surface area (230.0 cm^2^ g^−1^) and pore volume (0.2 m^3^ g^−1^). More importantly, the scaffold exhibited excellent apatite-forming ability and cellular biocompatibility. We believe that the hollow mesoporous bioactive glass nanofiber scaffold has great potential application in bone tissue regeneration.

## 1. Introduction

Mesoporous bioactive glasses (MBGs) have attracted extensive attention in clinical applications due to their excellent in vitro bioactivity [1,2,3,4,5,6], bone conductivity [7], and bone inducibility [8]. Compared to traditional bioactive glasses (BGs), MBGs exhibit large specific surface area and pore volume due to their well-ordered mesoporous structure. Yan et al. prepared MBGs and observed the MBGs possessed excellent bone-forming bioactivity compared with conventional BGs [9]. Li et al. fabricated multifunctional mesoporous silica nanoparticles and the multifunctional theranostic nano-platform for simultaneous photoluminescence imaging [10]. Kim et al. synthesized mesoporous bioactive glass nanoparticles to deliver therapeutic molecules, and the in vitro bioactivity test indicated their excellent apatite-forming ability [11]. However, the current research mainly focuses on MBG nanoparticles [12]. Bioactive glass nanofiber scaffolds have been recently reported, but their fiber diameter is on the micron scale, whereas the collagen fibers in a natural extracellular matrix (ECM) are between 50 and 500 nm in diameter [13]. Consequently, the structural similarity between glass fibers and the ECM is low.

Generally, an MBG nanofiber scaffold with higher similarity to the ECM is commonly prepared by the electrospinning technique, but electrospinning is complex, and the diameters of the obtained fibers are a few hundred nanometers [14,15]. Besides, as the fiber thickness is also on the micron scale, creating real-life biomimetic ECM structures is extremely challenging [14,16,17,18,19]. As a result, MBG hollow fibers with nanoscale diameters are almost never reported.

Bacterial cellulose (BC) has been widely applied as the scaffold in bone tissue engineering as its three-dimensional (3D) spatial network structure closely mimics the ECM structure [20,21,22]. Moreover, BC is increasingly used as the template in the preparation of inorganic materials [23,24]. However, the synthesis of MBG nanofiber scaffolds (especially those with hollow structures) on BC templates is rarely reported.

Motivated by these considerations, the present authors prepared a novel 3D hollow MBG nanofiber scaffold composed of hollow ultrafine MBG nanofiber scaffold (ca. 36 nm) via sol–gel assisted heat treatment technology, which was the smallest among all currently reported MBG fibers [25,26]. The sol-gel process is a low-temperature process employed to prepare MBG. Hydrolysis of the alkoxysilane groups is followed by condensation to form the glass network. A hybrid material can be obtained by introducing a polymer into the sol-gel process. The silica network develops in the dissolved polymer, thus allowing the silica network and the polymer to interact at the molecular level. In this preparation, the template was BC, and the pore-foaming agent was nonionic three-block copolymer P123 (polyethylene oxide–polypropylene oxide–polyethylene oxide). The morphology and structure of the 3D hollow MBG nanofiber scaffold were characterized by scanning electron microscopy (SEM), transmission electron microscopy (TEM), X-ray diffraction (XRD), N_2_ adsorption–desorption isotherm analysis, and nuclear magnetic resonance (NMR). The in vitro bioactivity and cellular biocompatibility of the MBG nanofiber scaffold were also investigated.

## 2. Results

### 2.1. Synthesis of Hollow Mesoporous MBG Nanofiber Scaffold

**Figure 1:**
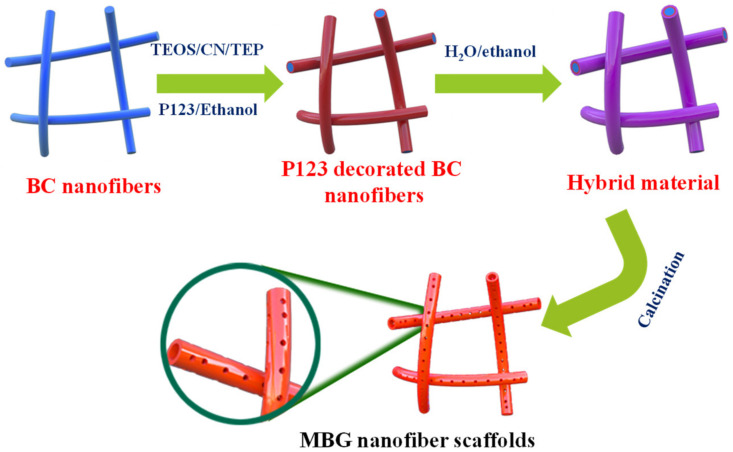
Schematic illustration of the synthesis of MBG nanofiber scaffold.

The synthetic procedure is shown in Figure 1. A hollow MBGs nanofiber scaffold was synthesized using BC as a template. Firstly, the BC template was immersed into P123/anhydrous ethanol solution for 24 h and then introduced to H_2_O/anhydrous ethanol solution for 24 h to complete hydrolysis and polycondensation. Finally, the 3D hollow mesoporous bioactive glass nanofiber scaffold was obtained using the sol-gel method and heat treatment technology.

### 2.2. Physicochemical Properties of the MBG Nanofiber Scaffold

**Figure 2:**
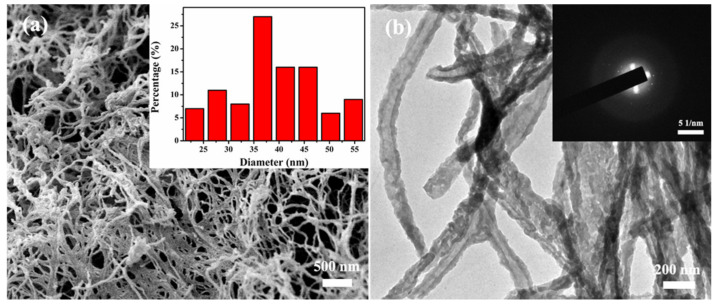
(**a**) SEM image and TEM image of MBG nanofiber scaffold (Inset shows the diameter distribution (**a**) and SAED pattern (**b**) of MBG nanofiber scaffold).

SEM and TEM images of the MBG scaffold are shown in Figure 2. The MBG scaffold is composed of nanofibers forming an obvious 3D spatial network with large interconnected pores (Figure 2a). This structure well replicates the structural characteristics of the BC template, so it is expected to promote cell proliferation, nutrient delivery, and bone growth [27,28]. Based on the SEM image, the diameter of the nanofiber scaffold is measured, and the average fiber diameter of the scaffold is approximately 36 ± 10 nm. The structural characteristics of the nanofibers were further analyzed by TEM. The MBG scaffold presents a large number of nanotube structures with uniform wall thickness (Figure 2b), which are advantageous for loading growth factors or drugs. The selected area electron diffraction (SAED) pattern (Figure 2b, inset) is a large spot, indicating an amorphous structure of the MBG scaffold. This phenomenon indicates that the inorganic components coated on the surface of the BC nanofibers were not crystallized after holding at 600 °C for 5 h and maintained their amorphous structure.

**Figure 3:**
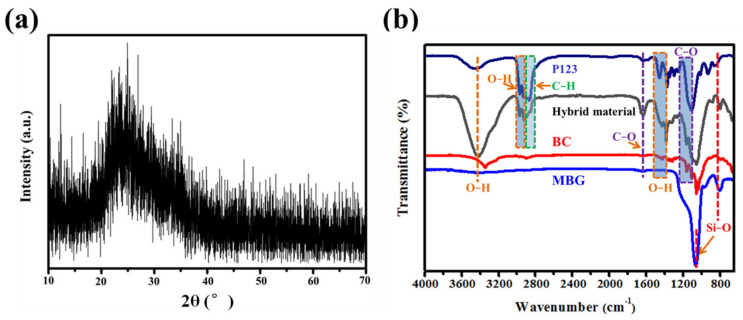
(**a**) XRD patterns and (**b**) FT−IR spectra of P123, BC, hybrid material, and MBG nanofiber scaffold.

The crystal structure of the MBG scaffold was determined from the XRD patterns shown in Figure 3a. The wide-angle diffraction peaks of the MBG scaffold are broad, with no obvious crystallization peaks, again indicating the amorphous nature of the MBG scaffold after high-temperature calcination. This result consolidates the SAED result (Figure 2b, inset). The wide SiO_2_ peak at 2θ ≈ 23° can be attributed to the amorphous silica. In the FT–IR spectrum of P123, BC, and hybrid material (Figure 3b), the major characteristic peaks of BC (3345 and 1493 cm^−1^ to O−H, 1164 and 1060 cm^−1^ to C−O) and P123 (2980–2850 cm^−1^ to C−H, 3000 cm^−1^ to O−H) is depicted in the FT−IR spectra of hybrid material. This clearly indicated the formation of P123 decorated BC nanofibers, hybrid materials, and calcination. For the MBG scaffold, the absorption peak at 1493 cm^−1^ is attributed to the hydroxyl group (−OH), and the characteristic peaks at 805 and 1040 cm^−1^ can be assigned to stretching and bending vibrations of the Si–O–Si bond, respectively [29,30].

**Figure 4:**
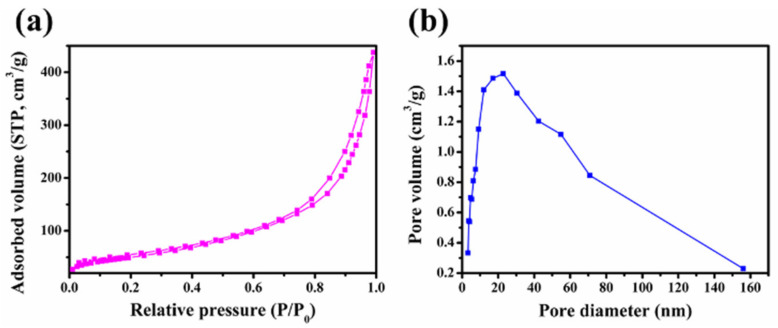
(**a**) N_2_ adsorption-desorption isotherms and (**b**) pore size distribution curves of MBG nanofiber scaffold.

Figure 4 displays the N_2_ adsorption–desorption isotherm and pore size distribution curve of the MBG scaffold. The sorption isotherm (Figure 4a) is a type IV isotherm with a B-type hysteresis loop in the 0.6–1.0 range of relative pressures caused by capillary agglutination. According to this result, the MBG scaffold is a typical mesoporous material [31,32]. The mesopore distribution curve of the MBG scaffold (Figure 4b) exhibits a high cusp distribution concentrated around 22.0 nm. The specific surface area, pore size distribution, and pore volume of the MBG scaffold, calculated using the Brunauer-Emmett-Teller (BET) and Barrett-Joyner-Halenda methods, are shown in Table 1. The MBG scaffold has a large specific surface area (230.0 m^2^ g^−1^) and a total pore volume of 0.2 cm^3^ g^−1^, implying a porous structure of the MBG scaffold. Such high porosity and large specific surface area will promote the loading and slow release of growth factors or drugs.

**Figure 5:**
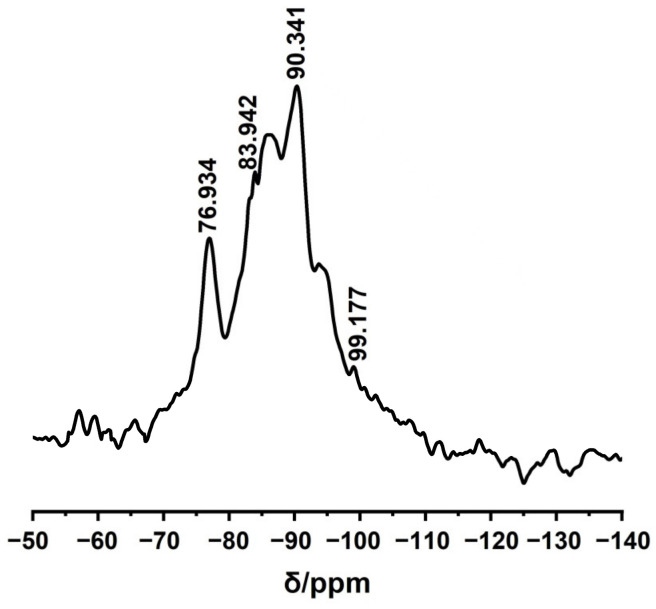
^29^Si NMR spectra of MBG scaffold.

Figure 5 shows the ^29^Si−NMR spectrum of the MBG scaffold. The obvious absorption peaks around chemical shifts of 83 and 90 ppm correspond to Si(OSi)_2_(OH)_2_ (Q^2^) and Si(OSi)_3_(OH) (Q^3^) in the silicon-oxygen network structure, respectively. Specifically, silicon contains two and one non–bridging oxygen bond at chemical shifts of 83 and 90 ppm, respectively. The additional weak peaks near chemical shifts of 76 and 99 ppm can be attributed to Si(OSi)(OH)_3_ (Q^1^) and Si(OSi)_4_ (Q^4^) in the silicon–oxygen network structure, respectively, which contain three and one non-bridging oxygen bonds, respectively. The above results reveal numerous non-bridging oxygen bonds in the MBG scaffold, which are generated by the breakage of the Si–O–Si network after introducing the basic metal ions [33]. The glass network structure also contains many non–bridging oxygen bonds, which can improve ion dissolution in the MBG scaffold, the degradation performance of the MBG scaffold, and the capacity of in vitro apatite formation. These results imply excellent in vitro biological activity of the MBG scaffold.

## 3. Discussion

### 3.1. Formation Mechanism of Hollow MBG Nanofiber Scaffold

Based on previous studies and reports [34], the authors suggest the following formation mechanism of the hollow MBG nanofiber scaffold. First, the Si, Ca, and P precursor solution and P123 were evenly dispersed in the ethanol solution. As no hydrolysis reactions occurred between the precursors and water during stirring in the sealed state, SiO_2_ nanoparticles could not form in the solution of precursors in ethanol. Dissolution in ethanol reduced the concentrations of TEOS, CN, and TEP before the BC was soaked in the mixture. As BC, with its 3D network structure, has excellent hydrophilicity, the precursor solution rapidly permeated the internal and external surfaces of the BC template and reacted with the −OH groups on the nanofiber surfaces, forming Si–O bonds via the in situ silicification reaction of the TEOS precursor (as shown in (1)). This reaction produced uniform (OR)_3_≡Si- deposits on the BC nanofibers. The strong polarity of the symmetrically structured (OR)_3_≡Si- promoted the adsorption of the other precursors, which underwent partial hydrolysis reactions to ethanol. However, the preceding hydrolysis reactions were impeded in the current experiment since the solvent was ethanol. The majority of the precursors just adsorbed on the BC nanofiber surfaces. Adsorption lowered the precursor concentrations in the BC nanofiber network structure, causing the precursor solution to diffuse continuously around the BC nanofiber into the 3D network structure. Finally, the precursor concentrations reached dynamic equilibrium on the BC nanofiber surfaces.

Hydrolysis: Si(OR)_4_ + nH_2_O ⟶ (OH)_n_ Si(OR)_4−n_ + nROH (1)

Condensation: 2Si(OH)_4_ ⟶ (OH)_3_Si-O-Si(OH)_3_ + H_2_O (2)

When the precursor-absorbed BC aerogel was immersed in the mixture of water and anhydrous ethanol, the (OR)_3_≡Si- groups and absorbed precursors underwent progressive hydrolysis and polycondensation (as shown in (2)). Similar to the synthesis of silica nanotubes by Hanagata [35], hydrolysis and polycondensation of the precursors produced a large number of colloid particles in the sol system. The BC nanofibers provided sites for the nucleation and growth of sol-gel-derived precursor oligomers, which were deposited on the BC nanofiber surface to a certain thickness along the axial direction. Eventually, a layer of amorphous precursor mixture was formed on the BC nanofibers. Over a prolonged soaking time, the concentration of the P123 surfactant exceeded the critical micelle concentration, inducing self-assembly in the Si, Ca, P, P123, and BC systems. At this time, mesoporous structures were formed on the inner and outer surfaces of the BC nanofibers. After sufficient hydrolysis, the BC nanofibers and P123 template were completely calcined, affording the MBG nanofiber scaffold with an ordered mesoporous structure and nanotube morphology.

### 3.2. In Vitro Bioactivity of the MBG Nanofiber Scaffold

**Figure 6:**
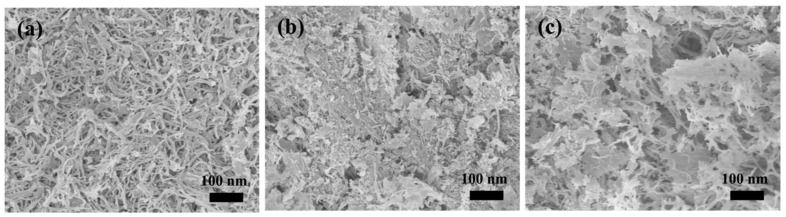
SEM images of MBG scaffold after immersion in SBF for (**a**) 12 h, (**b**) 24 h, and (**c**) 72 h.

Figure 6 displays the SEM images of the MBG scaffold mineralized in SBF at different times. Mineralization obviously changed the morphology of the MBG scaffold from that of the pre-mineralized sample (Figure 2a). After mineralization, a flaky mineral compound was deposited on the MBG scaffold’s surface, and the fibers were then attached. The mineral layer thickened as the soaking time was extended, and after 72 h of mineralization, the nanofibers were no longer discernible. The MBG scaffold’s huge surface area increased the SBF solution’s contact area and is anticipated to bestow superior in vitro biological activity. In particular, the large surface area enhances the dissolution velocity of the ions through the MBG scaffold and promotes the formation of silicon −OH groups on the scaffold surface, which act as nucleation sites for Ca and P ions.

**Figure 7:**
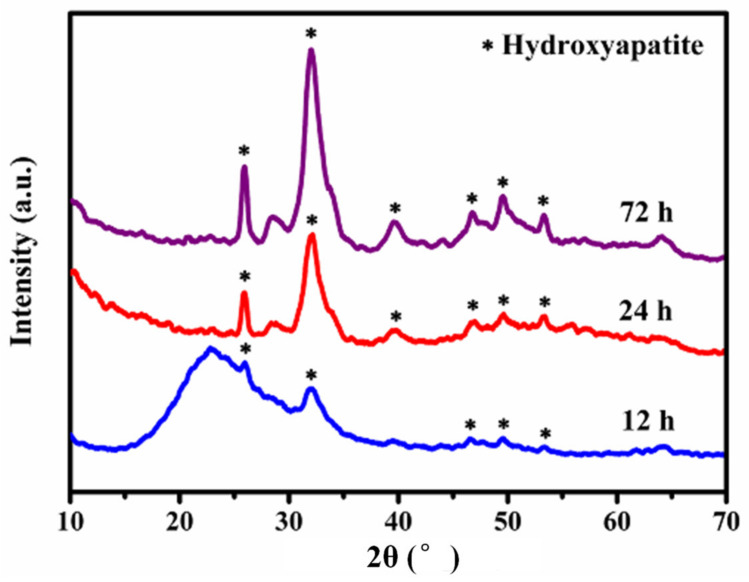
XRD patterns of MBG scaffold after immersion in SBF for different times.

Figure 7 displays the XRD patterns of the MBG scaffold mineralized in SBF at different times. After 12 h of mineralization, the XRD pattern of the scaffold showed not only amorphous peaks (Figure 3a) but also weak diffraction peaks (e.g., at 2θ ≈ 32°). Lengthening the mineralization time gradually increased the intensity of the peaks attributed to mineral formations on the MBG scaffold surface, and the crystalline state gained prominence. The peaks emerging at 2θ = 29°, 33°, 37°, 43°, 49.5°, and 53° were assigned to the (002), (211), (310), (222), (213), and (004) crystal planes of hydroxyapatite [36], respectively. These results affirm that the MBG scaffold can rapidly deposit hydroxyapatite in SBF and hence exhibits excellent in vitro biological activity. These results were consistent with those of the SEM and ^29^Si-NMR.

**Figure 8:**
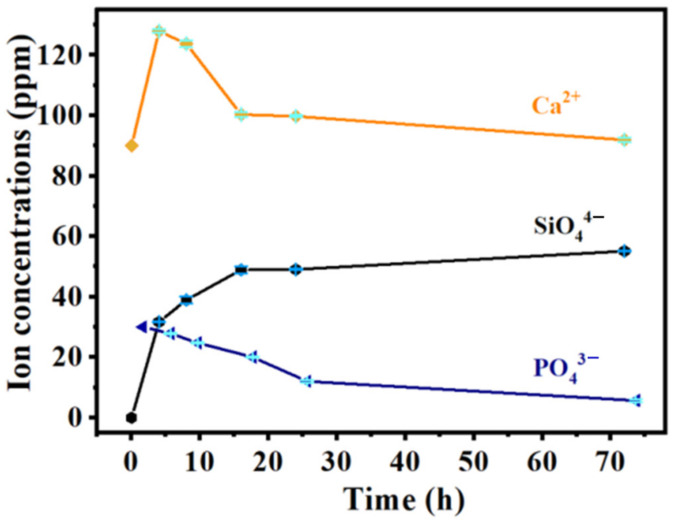
The curves of ion concentration released from MBG nanofiber scaffold for different time periods.

Figure 8 shows the release-concentration curves of ions (Ca^2+^, PO_4_^3−^ and SiO_4_^4−^) from the MBG scaffold at 4, 8, 16, 24, and 72 h. The Ca^2+^ concentration reached a maximum of 127.9 ppm after the first four hours and then gradually reduced until mineralization was complete. After 24 h of mineralization, the Ca^2+^ concentration reduced to 99.7 ppm. According to literature reports, the Ca^2+^ concentration change in SBF solution is related both to the dissolution of Ca^2+^ in the MBG scaffold and the formation of hydroxyapatite on the surface [37,38]. When the sample was immersed in SBF, the Ca^2+^ rapidly dissolved via hydrogen ion (H^+^) exchange in the solution. During the whole soaking process, the Ca^2+^ concentration in SBF would continuously decrease if the consumption rate of Ca^2+^ by hydroxyapatite exceeded the dissolution rate of Ca^2+^; otherwise, it would gradually increase. In the initial stage, the amount of Ca^2+^ released from the MBG scaffold exceeded the amount consumed by hydroxyapatite, so the Ca^2+^ concentration increased. At later times, the Ca^2+^ concentration reduced because ions were released from the MBG scaffold and many silica-rich gel layers formed on the surface of the MBG scaffold via silicon −OH groups. The gel layers provided nucleation sites for the Ca and P ions in the solution; consequently, the ions were deposited and crystallized on the gel surfaces. The consumption rate of Ca^2+^ then increased, and the Ca^2+^ concentration reduced. Meanwhile, the PO_4_^3−^ concentration trended downward in the SBF solution and decreased to 12.0 ppm after 24 h, mainly because PO_4_^3−^ was consumed by hydroxyapatite formation. In contrast, the SiO_4_^4−^ concentration trended upward because Si was released from the MBG scaffold.

### 3.3. Cellular Responses to MBG Scaffold

**Figure 9:**
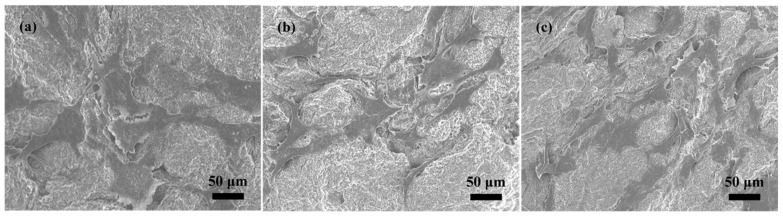
Morphology of the hBMSCs after culture on nanofiber scaffold for (**a**) 1, (**b**) 4, and (**c**) 7 days.

The attachment and morphology of hBMSCs on the MBG scaffold were examined by SEM (Figure 9). Many hBMSCs were observed to attach and spread well on the surface of the nanofiber scaffold after 1, 4, and 7 days, respectively. Besides, numerous filopodia were noted, demonstrating close cell contact with the scaffold. The result indicated the good biocompatibility of the MBG nanofiber scaffold.

## 4. Materials and Methods

### 4.1. Materials and Chemical

Pure ethyl alcohol, triethyl phosphate (TEP, 99.8%), calcium nitrate tetrahydrate (Ca(NO_3_)_2_.4H_2_O, 99%), and tertiary butanol were purchased from Tianjin Kemiou Chemical Reagent Co., Ltd. (Tianjin, China). Tetraethyl orthosilicate (TEOS, 98%) was purchased from Beijing Innochem Science & Technology Co., Ltd. (Beijing, China). Nonionic block copolymer EO_20_PO_70_EO_20_ (P123, Mw = 5800 g/mol) was purchased from Sahn Chemical Technology Co., Ltd. (Shanghai, China).

### 4.2. Preparation of MBG Nanofiber Scaffold

(1) First, 4 g of P123 was added to 50 mL of anhydrous ethanol and stirred until completely dissolved. To this solution, we successively added 9 mL of tetraethyl orthosilicate (TEOS), 1.75 g of calcium nitrate (CN), and 1 mL of triethyl phosphate (TEP). The obtained mixed solution was thoroughly stirred until it became uniform. Next, 25 mg of BC aerogel was soaked in this solution for 24 h. Once removed, the BC aerogel was washed twice quickly with ethanol and stored for later use.
(2) Second, 5 mL of H_2_O and 45 mL of anhydrous ethanol were combined and stirred to form an evenly mixed solution. The aerogel sample prepared in Step (1) was immersed in this solution for 24 h to complete hydrolysis and polycondensation. The obtained sample was freeze-dried to afford a SiO_2_/CaO/P_2_O_5_@BC hybrid material, which was then placed in a tubular furnace and heated from room temperature to 300 °C at 1 °C/min. After 30 min holding at 300 °C, the sample was heated to 600 °C and held at that temperature for 5 h, affording the targeted 3D hollow MBG nanofiber scaffold.

### 4.3. Characterization of MBG Nanofiber Scaffold

The microstructure of the scaffold material was observed using field-emission SEM with an acceleration voltage of 5 kV. The internal microstructure of the scaffold material was analyzed using field-emission TEM with an acceleration voltage of 200 kV. The wide-angle X-ray diffraction pattern of the sample was recorded by X-ray diffraction (XRD, Rigaku D/Max 2500 v/pc) with Cu Ka radiation. The chemical structure of the scaffold surface was measured with a Fourier transform infrared (FT–IR) spectrometer, and the surface area and pore size distribution of the scaffold were determined from the N_2_ adsorption–desorption isotherms. The ^29^Si NMR spectrum of the sample was recorded on an NMR spectrometer.

### 4.4. In Vitro Bioactivity Experiment of the MBG Nanofiber Scaffold

The scaffold was immersed in an appropriate amount of simulated body fluid (SBF). The mass: volume ratio of the material to the SBF was 1 mg mL^−1^. The SBF containing the scaffold was placed in an incubator rotating at 90 r min^−1^ at 36.5 °C for 12, 24, or 72 h. The SBF was refreshed every 24 h. The samples collected at different times were rinsed with deionized water, freeze-dried, and stored in sample bags for later use. Their surface morphologies and crystal structures before and after mineralization were characterized by SEM and XRD.

### 4.5. Cell Study

The hBMSCs used in this study were provided by Cyagen (China). The hBMSCs were then transplanted into culture flasks supplemented with human bone marrow stromal cell basal media (Cyagen, Taicang, China) and incubated at 37 °C in a humidified 5% CO_2_ incubator. Once the hBMSCs were passaged to about 80% confluence, the attached hBMSCs were further expanded. The medium was refreshed twice a week until the hBMSCs confluence was achieved. Only early passage cells were used in the next experiment.

To analyze the effect of the MBG scaffold on the cell morphology of hBMSCs, the cell/scaffold constructs were collected after being cultured for 1, 4, and 7 days in the growth medium, rinsed in PBS, and then fixed with 2.5% glutaraldehyde in PBS for 1 h. The construct was then removed by washing in PBS buffer containing 4% (*w*/*v*) sucrose and post-fixed in 1% osmium tetroxide in PBS. Then the constructs were dehydrated in a graded series of ethanol (50%, 60%, 70%, 80%, 90%, and 100% (*v*/*v*)) for 15 min. Finally, the scaffold was coated by spraying with Pt for 120 s. The morphologies of the attached hBMSCs were observed using SEM.

## 5. Conclusions

The repair of large bone defects is a challenging clinical problem. Limitations associated with existing treatments, such as autologous bone grafts and allografts, have increased the need for synthetic bone graft substitutes. This study aimed to investigate the in vitro bioactivity and cellular biocompatibility of novel 3D hollow MBG nanofiber scaffold as synthetic bone graft substitutes, which was successfully prepared from a BC template via the sol-gel-assisted heat treatment technology with P123 as the pore-forming agent. The obtained MBG scaffold revealed a 3D spatial network structure with an ultrafine tubular (around 36 nm) structure and possessed a large specific surface area of 230.0 m^2^ g^−1^ and a total pore volume of 0.2 cm^3^ g^−1^, implying a porous structure of the MBG scaffold. Additionally, the 3D MBG nanofiber scaffold showed good in vitro bioactivity, which was attributed to its large specific surface area. Cell study indicated the good biocompatibility of the MBG nanofiber scaffold, ascribed to the 3D spatial network structure. Therefore, this investigation demonstrated that the MBG nanofiber scaffold with excellent bioactivity and cellular biocompatibility would be a promising scaffold in bone tissue regeneration.

## Figures and Tables

**Table 1:** Specific surface area, pore volume, and nanopore size of MBG scaffold.

Samples	Specific Surface Area (m^2^ g^−1^)	Pore Volume (cm^3^ g^−1^)	Pore Size (nm)
MBG	230.0	0.2	22.0

## Data Availability

Not applicable.

## Sample Availability

Samples of the compounds MBG nanofiber scaffold and BC are available from the authors.
